# The *in Vitro* Antibacterial Efficacy of Persian Green Tea Extract as an Intracanal Irrigant on *Enterococcus faecalis* Biofilm

**DOI:** 10.22037/iej.2016.9

**Published:** 2016

**Authors:** Fatemeh Ramezanali, Shiva Samimi, Mohammadjavad Kharazifard, Farzaneh Afkhami

**Affiliations:** a * Department of Endodontics, Dental School, Tehran University of Medical Sciences, International Campus, Tehran, Iran; *; b * General Dentist, Tehran, Iran; *; c * Statistical Consultant, Dental Research Center, Tehran University of Medical Sciences, Tehran, Iran;*; d * Dental Research Center, Dentistry Research Institute, Tehran University of Medical Sciences, Tehran, Iran*

**Keywords:** Biofilm, *Enterococcus faecalis*, Green Tea Extract, Root Canal Irrigant, Sodium Hypochlorite

## Abstract

**Introduction:**

The aim of this study was to compare the antibacterial effect of Persian green tea extract (GTE) and 2.5% sodium hypochlorite (NaOCl) against *Enterococcus faecalis* (*E. faecalis*) as an intracanal irrigant.

**Methods and Materials:**

Thirty freshly extracted teeth were instrumented and sectioned into mesial and distal segments. The specimens were put into wells containing 2 mL of* E. faecalis*-containing medium. After 3 weeks, the specimens were removed and divided randomly into three groups (*n*=20). Each group was exposed to 3 mL of different irrigants for 3 min. Groups 1, 2 and 3 were irrigated with GTE, 2.5% NaOCl and normal saline, respectively. Biofilm formed in the middle third of the root canal was carved by sterile scalpel and cultured in Mueller-Hinton medium. Number of colony forming units (CFU) was counted on each plate. In addition, antimicrobial activity of the irrigants was evaluated by the agar disc diffusion test. The diameter of inhibition zone (IZ) around each irrigant was evaluated. The Kruskal-Wallis and Dunn tests were used to analysis the data.

**Results:**

While in NaOCl group no bacterial colonies were observed, the mean number of *E. faecalis *in GTE and control groups were 275±74 CFU/mL (*P*<0.001) and 119×10^8^±11×10^8^ (*P*<0.001), respectively. The mean of IZ in NaOCl and GTE groups were 24.35±0.78 and 6.9±0.87 mm, in order of appearance (*P*<0.001). Zone of inhibition was not observed around the control group (*P*<0.001).

**Conclusion:**

This research highlighted the potential role of plant extracts in antimicrobial root canal irrigation protocol.

## Introduction

Primary endodontic infections are due to existence of oral microorganisms which are present not only inside of the root canal system but also penetrate into the dentinal tubules. Moreover, some of them show resistance to endodontic antibacterial protocols. For example, *Enterococcus faecalis* (*E. faecalis*) is a resistant microorganism playing an important role in etiology of persistent endodontic infections [1]. Studies have shown that *E. faecalis* is the most commonly found microorganism in resistant lesions after root canal therapy [2, 3]. This bacteria is resistant to many of canal irrigants. Although mechanical and chemical preparations play an important role in disinfection of root canals, they are unable to completely remove all microorganisms from the complex areas of the root canal system. Thus, the need for use of antimicrobial agents for disinfection of the root canal system seems to be logical [4, 5].

There are many disinfectant solutions, with particular advantages and disadvantages [5]. For example, sodium hypochlorite (NaOCl) shows antimicrobial activity against a wide range of micro-organisms including *E. faecalis*, and also shows a relatively good tissue solubility. However this irrigant has several disadvantages such as cytotoxicity, tissue burning, bad taste and odor and discoloration of patient’s clothes [6, 7]. It has been shown that preventing direct contact between the periapical tissues and irrigants is impossible. Therefore, the use of solutions that are compatible to host tissues and show less tissue toxicity, especially if they also have anti-inflammatory properties can prevent unwanted inflammatory reactions in tissues around the roots [8]. Since these reactions do exist, many studies have focused on biological medicaments derived from plants to be used as an irrigant [9].

Plants have always been common sources of medical agents. Antimicrobial, antifungal and anticancer effects of many plants have been the subject of many studies [10-13]. In dentistry, herbal medicines have been used as anti-inflammatory, analgesic and antibacterial agents for many years [14-16].

Vinothkumar* et al.* [17] have examined the antibacterial effects of several herbal extracts including *Aloe barbadensis*, *myristica frangrans, terminalia chebula, curcuma longa *and* azadaricta indica* as endodontic irrigants. They used real-time quantitative polymerase chain reaction (qPCR) method, and the results of their study indicated the strong antibacterial property of plant extracts. Ghonmode *et al.* [18] studied the effect of *neem *leaf extracts and grape on *E. faecalis* and observed a very good antibacterial effect of these extracts. In a study by Naderi *et al.* [19], green tea and black tea extracts exhibited a marked antibacterial effect on* Streptococcus mutans*.

Persian green tea is obtained from the leaves of young *camellia sinensis* tree [19]. Its antibacterial activity is due to inhibition of the DNA gyrase bacterial enzymes by binding to the ATP binding sites of the ATP*B* subunit [20]. Green tea has antibacterial activity against *E. faecalis* and it also has been known as a chelating agent [21, 22].

The purpose of the present study was to compare the antibacterial effect Persian green tea extract (GTE) and 2.5% NaOCl against *E. faecalis* as intracanal irrigants.

## Materials and Methods

***Preparation of green tea extract***

The method of extracting green tea was as follows: 2 kg of Lahijan dried green tea (Tea Research Station of Lahijan, province of Guilan, Iran) was grinded in an electric mill (Braun, Model1021, Germany). The powder was then sieved using sieves with openings of 425 µm. The prepared powder was poured in two 2-L Erlenmeyer flasks (1 kg in each). Then, 1 L of 70% ethanol (Kimiagar, Tehran, Iran) was added to the Erlenmeyer flasks so that the entire dried surface of tea became wet with some extra ethanol covering over the tea powder. Erlenmeyer flasks were covered with aluminum foil sheets, and were put in oven at the temperature of 60^°^C for 72 h. Then the solution was filtered first through a 0.5 mm mesh strainer once, and then through a 0.1 mm mesh strainer twice. The volume of the solution at this stage was 750 mL. The ethanol was evaporated and the extracts were concentrated using rotary flask evaporator (Superfit, Mumbai, India) (8 circulations under vacuum conditions). The concentration discontinued at a stage prior to plasma formation since it was difficult to collect and inject plasma-shaped concentrate. Then the extract was heated indirectly in boiling water for 25 min.

***Assessment of anti-biofilm activity***

The protocol of the study was approved by the institutional review board (Grant No.: 2664). Thirty intact mature human mandibular premolars, free of anomalies, cracks or severe curvature that were extracted due to periodontal disease, were selected for this study. The teeth were kept in 0.5% NaOCl solution after extraction for 2 h for the purpose of cleaning and removal of organic debris. Then the teeth were kept in normal saline. 

The teeth were cut perpendicular to their long axis to obtain 8 mm roots. Canal working length (WL) was measured using a #15 K-file (Dentsply Maillefer, Ballaigues, Switzerland). The WL was determined 1 mm short of the file length after extrusion of the tip through the apical foramen. Each canal was prepared with ProTaper instruments (S1, S2, F1, F2, and F3, Dentsply Maillefer, Ballaigues, Switzerland) using crown-down technique according to the manufacturer's instructions. Canals were irrigated with 5 mL of 2.5% NaOCl during instrumentation. Then the roots were sectioned into mesial and distal halves along the mid-sagittal plan.

The specimens were then sterilized in a steam autoclave (Phoenix, Araraquara, SP, Brazil) for 15 min at 120^°^C and put into wells containing 2 mL medium inoculated with *E. faecalis* (ATCC 29212). For the purpose of biofilm formation under aseptic conditions, the plate was incubated in sterile conditions at 37^°^C and 100% humidity for 3 weeks and 1 mL of the culture medium was replaced with 1 mL of fresh medium every 48 h.

After 3 weeks, the teeth were removed from the wells and were divided randomly into three groups (*n*=20). Teeth in groups 1, 2 and 3 were put in 3 mL of GTE, 2.5% NaOCl and normal saline solution, respectively, for 10 min and then were washed with 10 mL of distilled water. 

Biofilm formed at the middle part of the root canal, approximately 1 mm thick, was cut off using a sterile scalpel blade and were cultured on Mueller-Hinton plates. The plates were incubated at 37^° ^C for 24 h and then, the number of colonies was counted.

***Agar diffusion test***

One hundred microliters of the 0.5 McFarland *E. faecalis* suspension were cultured on bile esculin agar plates. Wells of 3 mm diameter were made in the agar surfaces. Then 100 μL of GTE, 2.5% NaOCl and normal saline, were added to the respective wells and the plates were incubated for 24 h at 37^°^C in an incubator. After incubation period, plates were removed and the diameters of zone of inhibition (ZOI) were recorded. This procedure was carried out on 3 culture plates.

***Statistical analysis***

Statistical analyses were performed using the SPSS software (SPSS version 20.0, SPSS, Chicago, IL, USA). The Kruskal-Wallis test was used to compare the number of *E. faecalis* bacterial colonies, and Dunn test for comparing ZOI between the two experimental groups. The significance level was set at 0.05.

## Results

The results of antimicrobial activity of the groups are presented in Table 1. A reduction in CFU count was significant between NaOCl and GTE groups (*P*<0.0001). The control group showed 119×10^8^±11×10^8^CFU/mL which was significantly more than the experimental groups (*P*<0.05)

The mean of ZOI in 2.5% NaOCl group was 24.35±0.78 mm (ranging from 23 to 25 mm). The corresponding figures in GTE group were 6.9±0.87 mm (range 5.7 to 8.5 mm). The difference between the two groups was significant according to the Dunn test (*P*<0.0001). No ZOI was detected around the control group.

## Discussion

*E. faecalis *is the most commonly isolated species from the root canals of endodontically treated teeth with refractory or recurrent apical periodontitis [23]. Moreover, in dentinal tubules, bacteria surviving from chemical and mechanical cleaning may form biofilm and cause infection in the treated root canals [24].

When bacteria form a biofilm, the ensuing genetic and metabolic changes prevent entrance of antibacterial agents into the bacteria cell. Moreover, the antibiotic resistance increases compared to that of planktonic cells [25, 26]. Thus, studies on the effect of anti-bacterial agents on planktonic cells can not completely simulate the real *in vivo* situation. Therefore, the antibacterial effect of experimental irrigants on both *E. faecalis *colonies and its biofilm, were evaluated in this study.

It is clear that the power of biofilm formation and structural organization of biofilm is affected by the chemical nature of the substrate. Thus, formation of biofilm on other surfaces than dentine and root canals cannot create conditions such as what really exists on dentin and root canal walls [27]. Therefore, the formation of *E. faecalis* biofilm on tooth substrate in the present study was performed according to the methodology described by Kishen *et al.* [28].

The three-week incubation period in our study was based on previous studies showing that at this period the bacteria are able to penetrate up to 200-400 µm deep into the dentinal tubules [29]. Thus, different irrigants tested in this study were used in direct contact with the 4-week-old biofilms formed on tooth substrate.

It has been shown that the best solvent that can be used to collect crude extract of a plant is 80 or 85% ethanol or methanol since these solvents are able to solve up to 80% of the plant constitutes [30]. Moreover, disinfectants containing 70-79% ethanol are considered as the most effective disinfectants [31]. Therefore, ethanol was selected as a solvent to collect GTE in the present study.

In the present study, we found that GTE showed a good antibacterial activity against *E. faecalis* compared to the control group. Green tea has active materials with useful physiological effects. Its healing properties such as antioxidant activity, anti-inflammatory and radical scavenging properties are all useful characteristics making it appropriate for intra-canal irrigation [16]. It has been also used as a chelator agent [32]. Availability, affordability, low toxicity and long shelf life are some of the other good characteristics of green tea as a cleaning agent. No report of microbial resistance exists regarding this substance [33]. 

In the present study, NaOCl showed better antibacterial property against *E. faecalis* compared to GTE. NaOCl has burning properties and is a non-specific irrigant the action of which is not limited to necrotic tissues. It also has detrimental effects on dentin such as reducing its flexural strength and elastic modulus. It is used in a variety of concentrations from 0.5% to 5.25% for intra-canal irrigation [34]. Toxicity of NaOCl on vital periodontal tissues has been proved [35]. 

Several studies have demonstrated the antibacterial property of GTE against different bacterial species [33]. Rosaline *et al.* [21] used NaOCl, EDTA, saline, *morinda citrifolia, azadiracta indica* and green tea as a final irrigant. Significantly less bacterial adhesion was observed in samples treated with *neem*, NaOCl, green tea and *morinda citrofolia*, respectively.

**Table 1:** The mean (SD) of growth inhibition zone and number of colony forming units (CFU) of *E. faecalis*

**Groups**	***E. faecalis*** ** (CFU/mL)**	**Inhibition zone (mm)**
**Green tea**	275 (74)	6.9 (0.87)
**NaOCl**	0	24.35 (0.78)
**Control**	119×10^8 ^(11×10^8^)	0

Prabhakar *et* *al.* [22] compared the antimicrobial effects of green tea, Triphala, MTAD, and 5% NaOCl against* E. faecalis* and showed that 5% NaOCl was the most effective antibacterial agent. Triphala, green tea and MTAD also showed significant antibacterial effects. The researchers suggested the use of herbal irrigants inside the canal as an alternative to NaOCl to avoid detrimental properties of NaOCl. We also found better antibacterial efficacy in the NaOCl group, although GTE also showed acceptable antibacterial effects against *E. faecalis*. Some differences between the results of that study and our results might be due to the differences in the method of irrigation, the type of green tea, and culture time.

## Conclusion

Green tea showed acceptable antibacterial effect on the biofilm of *E. faecalis*. It seems that the use of herbal alternatives as intra canal irrigant should be considered to avoid detrimental properties of NaOCl perhaps in patients who are advocates of natural organic remedies.

## References

[1] Stuart CH, Schwartz SA, Beeson TJ, Owatz CB (2006). Enterococcus faecalis: its role in root canal treatment failure and current concepts in retreatment. Journal of endodontics.

[2] Gomes BP, Pinheiro ET, Jacinto RC, Zaia AA, Ferraz CC, Souza-Filho FJ (2008). Microbial analysis of canals of root-filled teeth with periapical lesions using polymerase chain reaction. J Endod.

[3] Siqueira JF, Jr (J Endod. 2008). , Rocas IN. Clinical implications and microbiology of bacterial persistence after treatment procedures.

[4] Mohammadi Z, Abbott PV (2009). Antimicrobial substantivity of root canal irrigants and medicaments: a review. Aust Endod J.

[5] Zehnder M (2006). Root canal irrigants. Journal of endodontics.

[6] Razmi H, Bolhari B, Dashti NK, Fazlyab M (2016). The Effect of Canal Dryness on Bond Strength of Bioceramic and Epoxy-resin Sealers after Irrigation with Sodium Hypochlorite or Chlorhexidine. Iranian endodontic journal.

[7] Mohammadi Z, Shalavi S, Giardino L, Palazzi F, Asgary S (2015). Impact of Ultrasonic Activation on the Effectiveness of Sodium Hypochlorite: A Review. Iranian endodontic journal.

[8] Giardino L, Estrela C, Generali L, Mohammadi Z, Asgary S (2015). The in vitro Effect of Irrigants with Low Surface Tension on Enterococcus faecalis. Iranian endodontic journal.

[9] Palombo EA (2011). Traditional Medicinal Plant Extracts and Natural Products with Activity against Oral Bacteria: Potential Application in the Prevention and Treatment of Oral Diseases. Evid Based Complement Alternat Med.

[10] Sahreen S, Khan MR, Khan RA, Shah NA (2013). Estimation of flavoniods, antimicrobial, antitumor and anticancer activity of Carissa opaca fruits. BMC Complement Altern Med.

[11] Kaschula CH, Hunter R, Parker MI (2010). Garlic-derived anticancer agents: structure and biological activity of ajoene. Biofactors.

[12] Mothana RA, Lindequist U, Gruenert R, Bednarski PJ (2009). Studies of the in vitro anticancer, antimicrobial and antioxidant potentials of selected Yemeni medicinal plants from the island Soqotra. BMC Complement Altern Med.

[13] Ashour HM (2008). Antibacterial, antifungal, and anticancer activities of volatile oils and extracts from stems, leaves, and flowers of Eucalyptus sideroxylon and Eucalyptus torquata. Cancer Biol Ther.

[14] Abbaszadegan A, Gholami A, Ghahramani Y, Ghareghan R, Ghareghan M, Kazemi A, Iraji A, Ghasemi Y (2016). Antimicrobial and cytotoxic activity of Cuminum cyminum as an intracanal medicament compared to chlorhexidine gel. Iranian endodontic journal.

[15] Shakouie S, Eskandarinezhad M, Gasemi N, Milani AS, Samiei M, Golizadeh S (2014). An in vitro comparison of the antibacterial efficacy of triphala with different concentrations of sodium hypochlorite. Iranian endodontic journal.

[16] Sinha DJ, Sinha AA (2014). Natural medicaments in dentistry. Ayu.

[17] Vinothkumar TS, Rubin MI, Balaji L, Kandaswamy D (2013). In vitro evaluation of five different herbal extracts as an antimicrobial endodontic irrigant using real time quantitative polymerase chain reaction. J Conserv Dent.

[18] Ghonmode WN, Balsaraf OD, Tambe VH, Saujanya KP, Patil AK, Kakde DD (2013). Comparison of the antibacterial efficiency of neem leaf extracts, grape seed extracts and 3% sodium hypochlorite against E feacalis - An in vitro study. J Int Oral Health.

[19] Naderi NJ, Niakan M, Kharazi Fard MJ, Zardi S (2011). Antibacterial activity of Iranian green and black tea on streptococcus mutans: an in vitro study. J Dent (Tehran).

[20] Gradisar H, Pristovsek P, Plaper A, Jerala R (2007). Green tea catechins inhibit bacterial DNA gyrase by interaction with its ATP binding site. J Med Chem.

[21] Rosaline H, Kandaswamy D, Gogulnath D, Rubin M (2013). Influence of various herbal irrigants as a final rinse on the adherence of Enterococcus faecalis by fluorescence confocal laser scanning microscope. J Conserv Dent.

[22] Prabhakar J, Senthilkumar M, Priya MS, Mahalakshmi K, Sehgal PK, Sukumaran VG (2010). Evaluation of antimicrobial efficacy of herbal alternatives (Triphala and green tea polyphenols), MTAD, and 5% sodium hypochlorite against Enterococcus faecalis biofilm formed on tooth substrate: an in vitro study. J Endod.

[23] Peciuliene V, Reynaud AH, Balciuniene I, Haapasalo M (2001). Isolation of yeasts and enteric bacteria in root-filled teeth with chronic apical periodontitis. Int Endod J.

[24] Delgado RJ, Gasparoto TH, Sipert CR, Pinheiro CR, Moraes IG, Garcia RB, Bramante CM, Campanelli AP, Bernardineli N (2010). Antimicrobial effects of calcium hydroxide and chlorhexidine on Enterococcus faecalis. J Endod.

[25] Socransky SS, Haffajee AD (2002). Dental biofilms: difficult therapeutic targets. Periodontol.

[26] Mah TF, O'Toole GA (2001). Mechanisms of biofilm resistance to antimicrobial agents. Trends Microbiol.

[27] McBain A, Gilbert P, Allison D (2003). Biofilms and biocides: Are there implications for antibiotic resistance?. Reviews in Environmental Science and BioTechnology.

[28] Kishen A, George S, Kumar R (2006). Enterococcus faecalis‐mediated biomineralized biofilm formation on root canal dentine in vitro. Journal of Biomedical Materials Research Part A.

[29] Kho P, Baumgartner JC (2006). A comparison of the antimicrobial efficacy of NaOCl/Biopure MTAD versus NaOCl/EDTA against Enterococcus faecalis. Journal of endodontics.

[30] Turkmen N, Sari F, Velioglu YS (2006). Effects of extraction solvents on concentration and antioxidant activity of black and black mate tea polyphenols determined by ferrous tartrate and Folin–Ciocalteu methods. Food chemistry.

[31] Christensen R, Robison R, Robinson D, Ploeger B, Leavitt R, Bodily H (1989). Antimicrobial activity of environmental surface disinfectants in the absence and presence of bioburden. Journal of the American Dental Association (1939).

[32] Lahijani S, Raoof Kateb H, Heady R, Yazdani D (2006). The effect of German chamomile (Marticaria recutita L) extract and tea tree (Melaleuca alternifolia L) oil used as irrigants on removal of smear layer: a scanning electron microscopy study. International endodontic journal.

[33] Abascal K, Yarnell E (2002). Herbs and Drug Resistance: Part 2-Clinical Implications of Research on Microbial Resistance to Antibiotics. Alternative & Complementary Therapies.

[34] Zehnder M (2006). Root canal irrigants. J Endod.

[35] Tanomaru Filho M, Leonardo M, Silva L, Anibal F, Faccioli L (2002). Inflammatory response to different endodontic irrigating solutions. International endodontic journal.

